# Prolonged Jaundice

**Published:** 1904-10-29

**Authors:** 


### PROLONGED JAUNDICE.

The clinical problem of a more or less prolonged jaundice is not a very infrequent experience in medical practice, and it is often attended with much difficulty in reference to diagnosis. Existing, as the jaundice in these instances may do, with few or no other obvious symptoms, there is little to guide the practitioner to a positive conclusion, and hence such cases are apt to cause stress apart from their strictly medical aspects. A frank confession of ignorance or uncertainty is a strain on faith and confidence which only the exceptional layman can be expected to sustain, whilst a precise and definite statement is felt by the physician to be in advance of the facts and to run the risk of a sharp negative from future events. This position has been rendered not less but more acute by the modern development of abdominal surgery. So long as no aid from this source came within the area of discussion, various schemes of medicinal treatment could be adopted in turn in the hope that, whatever the diagnosis, one or other of these would prove successful. But with the intervention of the surgeon as a recognised possibility, there soon arises in any case of prolonged jaundice the question whether or not it is justifiable or necessary to advise an operation, and the case therefore acquires a correspondingly increased anxiety. A mere theoretical knowledge would suggest that the differential diagnosis in the cases to which reference is now made cannot present any great difficulty. But practice and experience produce a very different conclusion, and often compel the practitioner to confess that whilst a mechanical obstruction is undoubtedly present in, or at the orifice of, the bile-duct, he can form no confident conclusion in regard to the nature of that obstruction.

Possibly the most immediate suggestions which arise in such a case are those of gall-stone and malignant disease. It is no doubt true that in the majority of cases of cancer of the liver jaundice is a comparatively late event, and merely tends to confirm a diagnosis which has already been suggested by the local physical signs and by the patient's general condition. But to this rule there are many exceptions, and jaundice may without question be the first, and for many months, the only, clinical evidence of malignant disease involving either the common bile-duct or the substance of the liver. Similarly, the jaundice due to an impacted gall-stone is in most instances associated with a history of previous temporary attacks which from the pain and other symptoms that accompany them are not difficult of interpretation. But here again exceptions occur. Thus, in a case reported by Chadwick,[1] there was only a solitary attack of biliary colic which was followed by jaundice, and the latter condition persisted without further change until the patient's death some six years later. An even more extreme exception was noted by Henry Morris.[2] The patient was a man of 69 years who, after jaundice of six months' duration without pain, tremor, or vomiting, was entirely relieved by cholecystotomy and by the extraction of 43 gall-stones. It is therefore evident that even the entire absence of the pain and other evidences of biliary colic is not an absolute proof that an existing jaundice is not due to gall stone. Such a conclusion does not lessen the difficulty created by the fact that not very rarely cancer of the liver exists without either pain or local tenderness. It may, indeed, be stated strongly that absence of pain decidedly opposes the suggestion of gall-stone; yet, as the case just quoted shows, this cannot be advanced as an unfailing guide. Such a condition, however, in a balanced diagnosis between malignant disease and gall-stone points very decidedly in favour of the former.

Another point worthy of attention when the diagnosis appears to lie between the two diseases just mentioned is the continuity of the jaundice. The fact that jaundice has been interrupted by more or less numerous intervals obviously favours a theory of gall-stone, and its persistence for several years has the same tendency, or at least such a record excludes cancer. But when the history shows a jaundice merely continued for several months either of the above explanations is possible, and to this may be added the remark that spontaneous cessation at any time leans decidedly in the direction of gall-stone. But even this is not conclusive. Mayo Robson[3] has drawn attention to the fact that malignant disease of the liver may be accompanied by catarrh of the biliary passages, and that this, together with the jaundice which it causes, may subside under treatment. Unless this possibility is borne in mind such an experience might prove very misleading, as disappearance of jaundice would, and in most cases rightly, strongly oppose a diagnosis of malignant disease A very exceptional case is recorded by Murchison,[4] in which, in an instance of malignant disease of the liver, jaundice, after existing for nine months, entirely disappeared, and remained absent for two months, when the patient died. The explanation of this very unusual occurrence, as discovered at the autopsy, was that the bile-duct had been blocked by a tumour mass which had sloughed, and had so allowed the bile to pass once again into the duodenum.

Taking next the physical examination of the hepatic region in a case of persisting jaundice, it has to be remembered that mere enlargement of the liver does not *per se* materially assist the diagnosis. Of course, if there is manifest nodulation, there is hardly room for doubt, but in many cases of cancer this is either absent or impossible of definition, and all that can be said with confidence is that the liver is to a greater or less extent enlarged. An impacted gall-stone is quite competent to produce the same result, and therefore mere increase in the size of the liver does not substantially advance the diagnosis. There is, however, one fact in the physical examination which in its positive relationship at least is of much significance. This is distension of the gall-bladder. Mayo Robson[3] attaches great importance to this as indicative of malignant disease, and, as is well known, he speaks on the basis of a very special experience. It may, indeed, be concluded that if there are exceptions to this rule they are very rare. Two very peculiar cases may here be noted, though as in neither of them was there jaundice they hardly invade the proposition just stated. In one[5] there were the physical evidences of an enlarged gall-bladder, and the patient after a sudden hæmatemesis suddenly vomited a large gall-stone. In the second[6] the patient complained of pain in the back and the epigastrium, and below the liver there was detected a movable lump which could be displaced downwards for several inches, this on operation was discovered to be a much elongated gall-bladder containing a single calculus of considerable size. But, as already indicated, such experiences hardly touch the general statement that in a case of persisting jaundice a definite enlargement of the gall-bladder affords very strong support to the view that the case is one of malignant disease. In reference to the physical examination of the hepatic region it is necessary to remark that an impacted gall-stone may produce very considerable changes in the liver and neighbouring structures. The hepatic congestion which attends such impaction and the peritoneal irritation and thickening caused by it may lead to such a sense of hardness and increased resistance, that a more serious condition than gall-stone may readily be suggested. All these considerations, therefore, go to show that there are many opportunities for error in connection with the physical facts in cases of obstinate jaundice of obscure causation, and that it is hardly possible to sustain any large general statement other than that already quoted, namely, that a manifestly distended gall-bladder indicates malignant disease rather than gall-stone.

Another series of symptoms to which considerable importance is attached in connection with impacted gall-stone is the occurrence at intervals of days or weeks of rigors, followed by extreme but short-lived outbursts of pyrexia, and accompanied, or not, by pain. Murchison[7] laid great stress on these, and more particularly on the fact that the jaundice became deeper after each of such attacks. But it is quite certain that very similar experiences may be met with in cancer, and C. O. Hawthorne[8] has published a series of cases showing the development of fever in malignant disease of the liver as well as in gall-stone, and in some of these there is a close correspondence between the temperature charts in the two conditions. Further, these intermittent febrile attacks were noted by Charcot,[9] who described them under the term *fièvre intermittente hépatique*, and who taught that such outbreaks were by no means confined to cases of gall-stone. On the contrary, he affirms that they may be found in any case where there is a prolonged or permanent obstruction in the bile-duct, as fibrous stricture, cancer of the head of the pancreas, etc. With this Murchison was in agreement, and even allows that he has seen such paroxysms "in two or three instances of catarrhal jaundice," though he contends that apart from gall-stone they are exceptional.

Reviewing all these considerations it is evident how difficult may be the explanation of a case of prolonged jaundice even when the diagnosis lies between gall-stone on the one hand and malignant disease on the other. In many instances, no doubt, there are facts in the case which make the physician's task a comparatively simple one. But in others a precise indication is altogether wanting, and every one of the lines of investigation above indicated must be thoroughly and carefully scrutinised and the evidence submitted to an impartial review.

But gall-stone and cancer are not the only possible causes of a prolonged jaundice, and hence in many instances the outlook must embrace other than these two diseases. Thus there are instances in which jaundice persists for several weeks or months, and yet ultimately disappears. It is usual to speak of these as cases of "catarrhal" jaundice, and probably this term is as applicable to them as any other which can be suggested. In other instances there are present with the jaundice marked evidences of febrile disturbance which may assume the intermittent type described by Charcot, and with each of these there may be considerable and even severe pain. The suggestion of gall-stone here is a very strong one; yet it has been made certain, in some of these cases, that gall-stone is not the explanation. An operation has, indeed, failed to detect any mechanical obstruction of the biliary passages, and it can only be surmised that some toxic agent has attacked the biliary, and perhaps also the pancreatic, tracts, causing among other things swelling of the mucous membranes, and a block to the escape of the bile. In one case[10] of this group, discharges of "membrane" from the bowel occurred, and there can be little doubt that the case was one of membranous enteritis. The fever, the pain, and the jaundice in these cases make their resemblance to gall-stone a very close one and the distinction may be impossible until either a calculus is passed on the one hand, or the negative result on opening the abdomen is exhibited in the other.

A further possibility as an explanation of long-continued jaundice is hypertrophic cirrhosis of the liver. In a case recorded by Sior[11] the jaundice persisted for nine months and the patient recovered under the administration of small and repeated doses of calomel. A still more exceptional case is one described by the late Sir George Johnson.[12] The patient was a woman of 39 years who had suffered from jaundice, soon followed by ascites, for 18 months. She was tapped on three occasions, and after the last of these the jaundice disappeared and, together with the ascites, remained absent for a period of five months. Both symptoms then returned and, in spite of treatment, the patient died soon after admission to hospital. At the autopsy there was discovered a fibrous thickening of the common bile-duct which obstructed its lumen just below the point where it is joined by the cystic duct. There was no evidence either of gall-stone or of syphilis, and the case, therefore, could only be reported as one of "simple" stricture. Probably the record example of prolonged jaundice is furnished by a case recorded by Dr. W. T. Cocking.[13] The patient was a woman of 50 years, and was said to have been jaundiced since she was three weeks old. The suggestion is that the condition was one of incomplete congenital stenosis, and the time of onset, together with the fact that the fæces were normal in colour, supports this view. Complete congenital stenosis or absence of the bile-ducts, with its accompanying jaundice, is invariably fatal, but it is conceivable that a partial stricture might be sufficient to interfere with the free flow of the bile and yet stop short of the fatal issue with which absolute obstruction in newly-born children is always associated. An interesting record in this connection is made by Dr. J. Glaister,[14] who relates a family history, including seven children, of whom "four had died jaundiced and two were less severely jaundiced and recovered." This family tendency in congenital absence or stenosis of the bile-ducts has been noted by Dr. J. Thomson of Edinburgh in his monograph on the disease, and it only remains to note as bearing on the case of jaundice of 50 years' duration above mentioned that a child of the patient died with signs of such obstruction in early infancy. This fact manifestly, in the cicumstances, lends some support to the suggestion that the cause of this life-long jaundice was a congenital though partial obstruction of the bile-duct.

[1] Brit. Med. Jour, 1895, Vol. I., p. 253. [2] The Operative Treatment of Gall-stones (Krauss). [3] On Gall-stones, and System of Medicine (Clifford Allbutt), Vol. IV. [4] Clinical Lectures on Diseases of the Liver, Third Edition. [5] Brit. Med. Jour., Sept. 9, 1893. [6] Lancet, Jan 5, 1901. [7] Ibid., 1879, Vol. I. [8] Brit. Med. Jour., March 16, 1901. [9] Maladies du Foie et des Reins. [10] Brit. Med. Jour., May 27, 1893. [11] Clin. Jour., Vol. II., p. 111. [12] Med. Lectures and Essays, p. 611. [13] Lancet, April 11, 1903. [14] Transactions Glasgow Medico-Chirurgical Society, Vol. I, p. 183

